# Geometry and Topology of Estuary and Braided River Channel Networks Automatically Extracted From Topographic Data

**DOI:** 10.1029/2019JF005206

**Published:** 2020-01-05

**Authors:** Matthew Hiatt, Willem Sonke, Elisabeth A. Addink, Wout M. van Dijk, Marc van Kreveld, Tim Ophelders, Kevin Verbeek, Joyce Vlaming, Bettina Speckmann, Maarten G. Kleinhans

**Affiliations:** ^1^ Department of Physical Geography, Faculty of Geosciences Utrecht University Utrecht Netherlands; ^2^ Department of Oceanography and Coastal Sciences, College of the Coast and Environment Louisiana State University Baton Rouge LA USA; ^3^ Coastal Studies Institute Louisiana State University Baton Rouge LA USA; ^4^ Department of Mathematics and Computer Science TU Eindhoven Eindhoven Netherlands; ^5^ Department of Information and Computing Science Utrecht University Utrecht Netherlands; ^6^ Department of Computational Mathematics, Science and Engineering Michigan State University East Lansing MI USA

**Keywords:** channel network extraction, estuaries, braided rivers, network analysis, estuarine geomorphology, fluvial geomorphology

## Abstract

Automatic extraction of channel networks from topography in systems with multiple interconnected channels, like braided rivers and estuaries, remains a major challenge in hydrology and geomorphology. Representing channelized systems as networks provides a mathematical framework for analyzing transport and geomorphology. In this paper, we introduce a mathematically rigorous methodology and software for extracting channel network topology and geometry from digital elevation models (DEMs) and analyze such channel networks in estuaries and braided rivers. Channels are represented as network links, while channel confluences and bifurcations are represented as network nodes. We analyze and compare DEMs from the field and those generated by numerical modeling. We use a metric called the volume parameter that characterizes the volume of deposited material separating channels to quantify the volume of reworkable sediment deposited between links, which is a measure for the spatial scale associated with each network link. Scale asymmetry is observed in most links downstream of bifurcations, indicating geometric asymmetry and bifurcation stability. The length of links relative to system size scales with volume parameter value to the power of 0.24–0.35, while the number of links decreases and does not exhibit power law behavior. Link depth distributions indicate that the estuaries studied tend to organize around a deep main channel that exists at the largest scale while braided rivers have channel depths that are more evenly distributed across scales. The methods and results presented establish a benchmark for quantifying the topology and geometry of multichannel networks from DEMs with a new automatic extraction tool.

## Key Points

- A new volume‐based method enables extraction of multithread channel networks from bathymetry across bed level jumps
- Both network topology and geomorphic information can be extracted at a range of spatial scales
- Estuaries typically form a single dominant channel at the largest spatial scale, while braided rivers show no scaling break

## 1 Introduction

Channels are ubiquitous features of Earth's surface that are important pathways for the transport of water, solids, and solutes across landscapes; provide a range of ecosystem services; and support economic activity. Channel patterns range significantly in complexity, from single‐thread, meandering rivers cutting across continents and the sea floor, to multithread channel systems that bifurcate and converge in braided rivers, estuaries, and deltas. These patterns exist over a range of spatial scales. Understanding and quantifying channel network patterns and geometry are vital precursors to predicting many important environmental processes including geomorphological change, water and sediment transport, and ecosystem dynamics. However, automated recognition of channels and their connections from bathymetry is not straightforward because most channel systems have large spatial and temporal variations in bed elevation, arrangement, and water depth.

**Figure 1:**
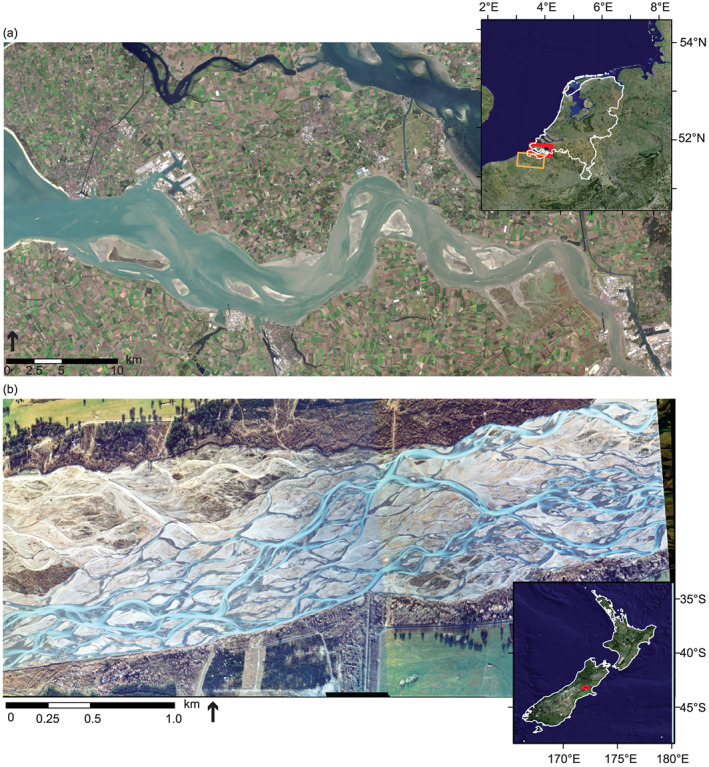
Examples of multichannel networks with similar frequencies of bifurcations and confluences: (a) The Western Scheldt Estuary in the Netherlands (LANDSAT 8 image downloaded from USGS Earth Explorer at https://earthexplorer.usgs.gov/) and (b) the Waimakariri River, a braided river north of Christchurch in New Zealand (imagery from Hicks et al., 2007). Inset images are composite satellite images produced by MDA Information Systems.

Quantifying patterns, structures, and geometries of channels is necessary to understand and predict landscape dynamics. Networks, which are mathematical representations of objects and the connections among those objects (Newman, 2003, 2010), are useful representations of topology and geometry in channelized systems (e.g., Benda et al., 2004; Czuba & Foufoula‐Georgiou, 2014; Dai & Labadie, 2001; Maidment, 2016; Marra et al., 2014; Rodriguez‐Iturbe & Rinaldo, 1997; Smart & Moruzzi, 1972; Tejedor et al., 2015a, 2015b). Generally speaking, three types of channel networks exist (Kleinhans, 2010; Limaye, 2017): (1) systems where flow paths are generally convergent, such as tributary stream networks with more frequent confluences than bifurcations; (2) systems with divergent characteristics like deltas and alluvial fans with more frequent bifurcations than confluences, and (3) chain‐like systems such as braided rivers, anastomosing rivers, and estuaries with similar frequencies of bifurcations and confluences (Figure 1). While methods relying on surface gradients are generally successful at extracting channel networks from topography in convergent systems (Passalacqua et al., 2015; Tarboton & Ames, 2001), the extraction of chain‐like, divergent and bifurcating channel networks from topographic data remains an open challenge. While progress has been made (e.g., Limaye, 2017;van Dijk et al., 2019), there is a need for an automatic method for the extraction and analysis of multithread channel network topology and geometry from topographic data. Consequently, we do not know and cannot quantify in what aspects the channel networks of braided rivers, deltas, and multichannel estuaries differ beyond the obvious. This paper aims to fill that gap. Results from earlier versions of this framework have been presented in van Dijk et al. (2019).

Channel networks are often identified from either digital elevation models (DEMs) (Fagherazzi et al., 1999; Montgomery & Dietrich, 1989; Passalacqua et al., 2015; Tarboton et al., 1991; Tarboton, 1997) or imagery (Dillabaugh et al., 2002; Edmonds et al., 2011; Isikdogan et al., 2015, 2017; Marra et al., 2014; Passalacqua et al., 2013; Pavelsky & Smith, 2008). Classically, methods for extracting channel networks from DEMs have relied on the concepts of steepest descent, flow direction assignment, and the delineation of channels based on flow accumulation (e.g., Lacroix et al., 2002; Pelletier, 2004; Shelef & Hilley, 2013; Tarboton et al., 1991; Tarboton, 1997; Tarboton & Ames, 2001). With the advent of high‐resolution topography data from lidar (Tarolli, 2014), sophisticated channel network identification algorithms for high‐resolution data have emerged in recent years (Lashermes et al., 2007; Passalacqua et al., 2010; Pelletier, 2013; Sangireddy, Stark, et al., 2016). Methods relying on surface gradients and flow accumulation are generally effective in convergent systems like tributary networks but fail in multithreaded channel networks that bifurcate and recombine. Important reasons are that the condition of flow following the path of steepest descent is violated, bed steps with negative slopes are present at bifurcations and confluences, and channels may diverge over shallow bars, shoals, and sills which renders their recognition with local path‐seeking algorithms impractical. These methods are also sensitive to noise and local highs. An alternative strategy for delineating channel networks from DEMs is through the use of hydrodynamic modeling to track inundation patterns. This strategy can robustly capture bifurcations and convergences in a complicated system (e.g., Limaye, 2017) but currently does not yield a network topology, while it can be computationally expensive and is sensitive to assumptions in boundary conditions and hydraulic resistance.

The identification of channels from imagery often requires the use of spectral thresholding or classification schemes to distinguish between water and land features, followed by mapping of channels from the resulting image in both the experimental (e.g., Ashworth et al., 2006; Wickert et al., 2013) and natural settings (e.g., Edmonds et al., 2011; Marra et al., 2014; Passalacqua et al., 2013; Welber et al., 2012). In numerical models generating multithread systems, thresholds are often used to distinguish channels from bars and floodplains (e.g., Liang et al., 2016; Schuurman & Kleinhans, 2015). More sophisticated algorithms exist (Dillabaugh et al., 2002; Isikdogan et al., 2015, 2017; Pavelsky & Smith, 2008), but current methodologies are sensitive to local bed elevation increases and still struggle to maintain channel network connectivity at bifurcations and confluences (Isikdogan et al., 2015).

Channel planform geometry is influenced by a plethora of environmental factors including water discharge (Leopold & Wolman, 1957; Van den Berg, 1995), sediment composition and transport (Braat et al., 2017; Church, 2006; Orton & Reading, 1993), lithology (Nittrouer et al., 2011; Townend, 2012), bank strength and vegetation (Millar, 2000; Tal & Paola, 2010; Tal et al., 2004; Vandenbruwaene et al., 2011), climate (Phillips & Jerolmack, 2016), and receiving basin characteristics like tides and waves (Galloway, 1975; Geleynse et al., 2011; Jerolmack & Swenson, 2007; Nienhuis et al., 2018; Rossi et al., 2016). Braided rivers have high rates of morphological change, which is due to the abundance of noncohesive sediment and high stream power (e.g., Hicks et al., 2007; Kleinhans & van den Berg, 2011). The primary requirements for the development of braided river patterns are thought to be the presence of a movable bed and a wide braid plain (Kleinhans, 2010; Kleinhans & van den Berg, 2011), although modeling work also suggests that bank erosion and boundary condition fluctuations are necessary for maintaining dynamic equilibrium (Schuurman et al., 2013). Estuarine channel network morphology is shaped by the competition between tidally and fluvially driven transport (Robinson, 1960; van Veen, 1950; Van der Wegen & Roelvink, 2012) and sediment composition (Braat et al., 2017). While subject to different boundary conditions, braided rivers and estuaries can share similar chain‐like multichannel networks that bifurcate and recombine at similar frequencies (Figures 1a and 1b). Thus, an investigation into the similarities and differences in channel network structures of estuaries and braided rivers may yield insight into the processes affecting their morphologies.

This paper introduces a mathematically rigorous and practical method for extracting multichannel networks from topographic data of rivers and estuaries in the field, numerical models, and physical experiments in order to analyze the structure and geometry of the channel network. The channel extraction tool, called LowPath, utilizes an algorithm first introduced by Kleinhans et al. (2019) that relies on identifying sets of channels that have the lowest path (in terms of elevation) from the source to the sink of the system. This framework produces a full network topology with geometric attributes using only topographic information and a scaling parameter related to a characteristic sediment volume. Comparisons are made between estuaries with multiple channels and braided rivers from both nature and morphodynamic numerical models. The output from these analyses yields insight into the processes affecting the morphology of multichannel systems.

The remainder of this paper is organized as follows. Section 2 provides an overview of the network extraction method, LowPath, and details the location of our modeling setup of four case studies: the Western Scheldt estuary in the Netherlands, Waimakariri River in New Zealand, the braided river model of Schuurman et al. (2013), and the multichannel, bar‐built estuary model of Braat et al. (2017). The results of the network extraction are presented in section 3, followed by results of the topological and geometric analyses performed on the extracted channel networks (section 4). The implications of the results are discussed in dection 5, along with an exploration of the role of scale in network delineation from topographic surfaces. Section 5 also contains notes for future avenues of research. The conclusions are stated in section 6.

## 2 Background and Methods

### 2.1 A Primer on Network Terminology

**Figure 2:**
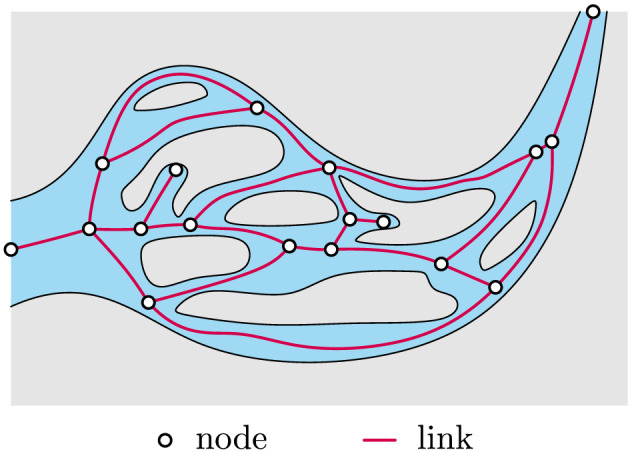
Example of a network for a multichannel system.

A network is a mathematical representation of a set of objects and the connections among those objects (Newman, 2003). Networks are made up of two types of elements, *links* and *nodes*, where links delineate how nodes are connected to each other. The mathematical representation of the interconnectedness in a network is called the network *topology*, which can be represented by an adjacency matrix where rows and columns represent nodes and the entries of the matrix represent the links between the nodes. In the case of a braided river or estuary, nodes represent bifurcations, or sometimes polyfurcations, confluences, inlets, and outlets, while links represent channel centerlines or thalwegs (Figure 2). A path is a sequence of links that connect the starting and ending nodes of the system.

### 2.2 Theory

A common challenge in geomorphology and hydrology is delineating a channel network from a DEM, due to complications such as longitudinal variations in channel depth and slope, and violations of steepest‐descent principles, among others. Recently, however, a framework called LowPath was introduced by Kleinhans et al. (2019) for the extraction of multithreaded channel networks from topographic surfaces. Here we describe the theory behind the operation of the LowPath algorithm. A detailed description of the mathematical principles underlying the method, as well as mathematical proofs, can be found in the work ofKleinhans et al. (2019). Additional details regarding LowPath operation and parameterization can be found in the supporting information.

The algorithm takes as input a DEM of the bed level of a braided river or estuary. Because it uses only the elevation of the bed level, the generated network is independent of the water level. However, the algorithm could in principle be applied to other maps, including depth or velocity fields.

The basic building block of a network generated by LowPath is the concept of a *lowest path*. Intuitively speaking, the lowest path between points s and t in the DEM is the path, consisting of edges of the DEM, that does not traverse any elevations higher than necessary to connect s and t. More formally, given two paths $p_{1}$ and $p_{2}$, we use the following procedure to check if $p_{1}$ or $p_{2}$ is *lower*. First, we check the elevations of the highest points of $p_{1}$ and $p_{2}$. If the highest point of $p_{1}$ is lower than that of $p_{2}$, then $p_{1}$ is lower (and vice versa). If their elevations are identical, we do the same for the second highest points of $p_{1}$ and $p_{2}$, and if those are identical again, we check the third highest points, et cetera. The lowest path from s to t is the path such that no lower path from s to t is possible.

Steepest descending paths have often been used for constructing drainage networks in tributary systems and generally rely on “pit‐filling” algorithms to remove any ascending topography that interrupts downslope flow accumulation (Tarboton, 2014). The lowest paths identified in LowPath consist of both descending and ascending parts (i.e., no pit filling is used). However, each descending piece is (part of) a steepest‐descending path, and each ascending piece is a steepest‐descending path in reverse. This property has been proven formally in Kleinhans et al. (2019) and is used by LowPath to compute lowest paths efficiently. For this purpose, the algorithm first computes a so‐called *descending Morse‐Smale complex* (MSC) (Edelsbrunner et al., 2001; Kleinhans et al., 2019; Shivashankar & Natarajan, 2012). The MSC of a DEM is a topological complex that describes the structural elements of the terrain. It contains certain critical points of the DEM: local minima and saddle points (points that are a local minimum in one direction while being a local maximum in the other). Additionally, it contains any steepest‐descent paths (called *MS links*) from saddle points toward minima. To find the lowest paths between any two points s and t in the DEM, the algorithm first computes steepest‐descent paths from s and t to critical points and then searches for the lowest path consisting of MS links between those critical points.

**Figure 3:**
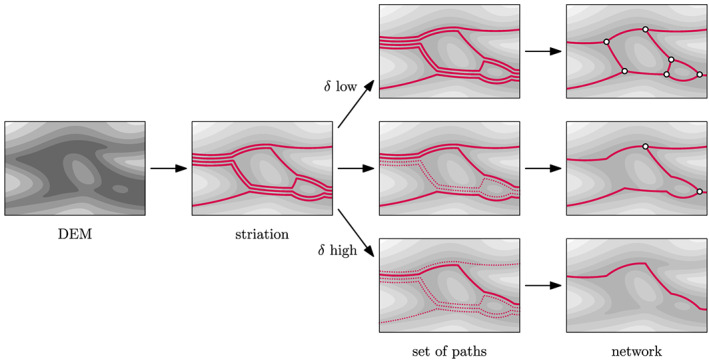
A qualitative depiction of how LowPath determines the channel network. First, from the river bed DEM, the striation is computed (left). Consequently a set of sufficiently different paths is found (center; here depicted for three values of the volume parameter δ), which form the final network (right).

Instead of just one single lowest path, the algorithm needs to compute a complete set of paths that together form the entire channel network. To achieve this, the algorithm sequentially finds lowest paths in parts of the DEM. More precisely, LowPath starts by computing a lowest path $p_{\text{lowest}}$ between two designated points on the boundary of the DEM (the “source” and the “sink”). After $p_{\text{lowest}}$ has been found, the DEM is split around $p_{\text{lowest}}$ into two parts. Then the same procedure is repeated: in both parts of the DEM, lowest paths are found, and then the DEM is split again around these paths. This procedure is repeated until no critical points are left in each part of the DEM. (In fact, the splitting procedure is somewhat more complicated, to avoid issues if the lowest path lies entirely on the boundary of its part of the DEM, in which case splitting along that path would not make the resulting DEM part smaller, hence resulting in an infinite loop. To avoid this, LowPath splits along not one but two parts at a time, thereby ensuring progress. We refer to Kleinhans et al., 2019, for more details.) All the paths found in this fashion form an ordered set of noncrossing paths, called a *striation* (see Figure 3, left).

In general the striation contains a large number of paths. Since the resolution of a DEM typically is such that channels are several to many grid cells wide, it may contain several paths within the same channel, which would be undesirable in the network. To alleviate this, we need a way to determine for two striation paths whether they are “sufficiently different” to form two separate channels. Then, the algorithm picks a subset of the striation paths, which are all sufficiently different, to obtain the network (see Figure 3, center and right).

To decide if two paths are sufficiently different, we consider the volume of sediment that separates them: The larger the volume, the more different the channels are. The sediment volume is a morphologically meaningful way to distinguish channels, because volume is related to the morphological work required to cut bars and merge channels (e.g., Kleinhans, 2005). However, since the elevation of paths can vary wildly, it is not immediately clear which volume to measure. For example, to measure the sediment volume between two paths $p_{1}$ and $p_{2}$, we might measure all the volume above the highest elevation on $p_{1}$ and $p_{2}$. However, this may underestimate the amount of sediment, for example, if some point on $p_{1}$ or $p_{2}$ has high elevation while the remainder of the paths is low. Similarly, if we measure the volume above the lowest elevation on $p_{1}$ and $p_{2}$, we likely overestimate the amount of sediment. To solve this, we would like to measure the volume of just the sediment that forms a barrier between the two paths. This is formalized mathematically by the concept of an *isotopy*, which is a smooth “morph” from $p_{1}$ to $p_{2}$. We take the isotopy that induces the smallest volume of sediment. In other words, we measure the minimum volume of sediment that obstructs one path from sliding downhill toward the other path (further detailed in supporting information Figure S2).

Given this method of determining the volume of sediment between paths, we define two paths to be sufficiently different, and allow them to be in the network together, if and only if this volume is larger than some *deposited sediment threshold* δ. We call this threshold the volume parameter (following the “sand function” presented in Kleinhans et al., 2019). Lowering δ means that channels with smaller bars in between are distinguished as sufficiently different channels. Higher δ values on the other hand require larger bars between channels for them to be distinguished as sufficiently different. The δ value associated with an identified channel represents the volume of sediment separating the channel from others in the network and is thus a representation of the spatial scale at which a channel exists (i.e., bigger channels tend to have large δ values). Therefore, by generating several networks with different values of δ, channels across a range of sizes are identified (Figure 3).

In the resulting network, the existence of a path can be affected by the existence of another path in seaward (downstream) or landward (upstream) direction. This is the result of the threshold δ being reached by the summation of several bar deposits between paths. This means that the threshold volume could in principle be reached by the volume at one end of the system alone depending on the order of the sorted paths. Therefore, for example, two paths in the network may be close to one another in one section of the system, simply because they are separated by a large volume of sediment in another section.

### 2.3 Workflow

The three main steps in the workflow are the preparation of the DEM, application of the LowPath algorithm as described in the previous section, and the assignment of topographic and geometric information to links and nodes. Preprocessing and postprocessing was performed in Matlab (MATLAB, 2017). Input data along with example processing scripts are available in Hiatt (2019).

We utilize LowPath version 1.3.6 which is part of the software package Topological Tools for Geomorphological Analysis (TTGA) (Sonke, 2019). As input, LowPath implementation requires only a topographic surface (image file or text file) to output the set of lowest paths and network nodes. Geometric properties of the DEM must be specified, including the horizontal resolutions of the grid cells in the x and y directions. Only rectangular grids are accepted, but the grid cells do not need to be square. The elevation range (i.e., the minimum and maximum elevations) of the DEM must be included for the image‐based input to be able to properly calculate volumes, because this is the best available estimate of the reworkable sand body that is assumed in the volume parameter. To ensure that only the river bed itself is analyzed for network paths, and not for example the surrounding floodplain or human–built features, as a preprocessing step the user is able to mask grid cells that are outside the domain of interest. The user must also specify the δ value or range of values.

As described in the previous section, LowPath generates a network consisting of a set of sufficiently different paths. How many paths are included in the network is determined by the selection of δ. At the higher end of the δ spectrum (i.e., large volumes of sediment) only a single path is extracted. This is the overall lowest path that traverses the riverbed. As δ decreases, the number of paths extracted generally increases, because the volume between adjacent paths needed to identify channels as sufficiently different is decreasing. Eventually, as δ nears 0, the returned network contains all striation paths. In other words, varying the parameter δ allows us to obtain networks across a wide range of scales.

**Figure 4:**
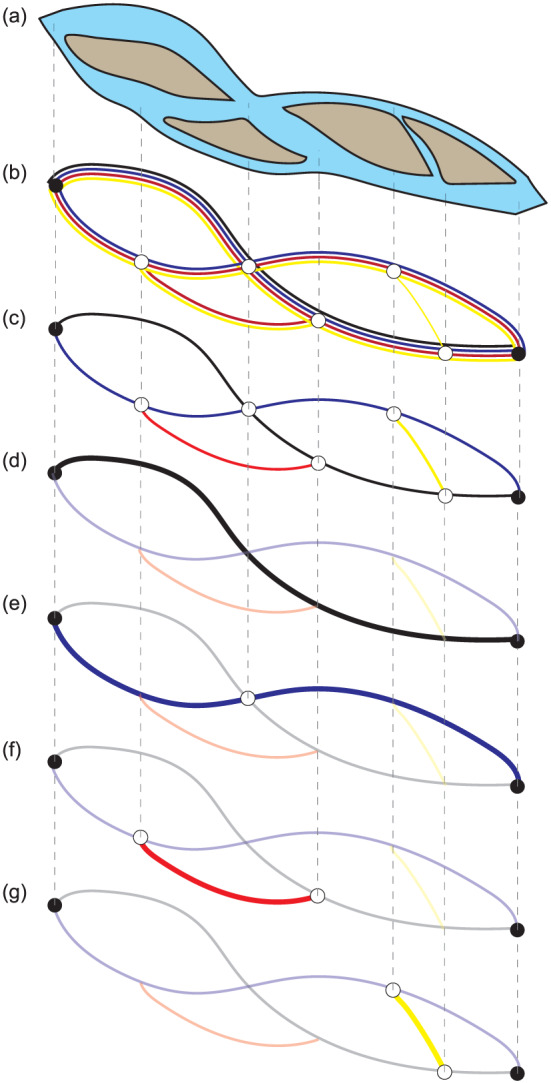
Breakdown of the steps necessary to create a network from topographic data (a) using LowPath and postprocessing tools. (b) Channel centerlines and locations of overlap or nodes (circles) are output from LowPath across a range of volume parameter scales (from smallest to largest scale: yellow, red, blue, and black). Adjacent lines depict overlap of channels extracted at different scales. The smallest scale channels are detected everywhere that a larger‐scale channel is also detected, leading to relatively large/deep channels being detected at a large number of scales (depicted by adjacent links). (c) Overlapping channels are systematically removed such that each detected channel centerline belongs to a single volume parameter scale. (d–g) Finally, the network is segmented into differential networks associated with a single volume parameter scale. Doing so allows channel geometries to be assigned to the network independent of the influence from other scales.

In this paper, we want to classify individual channels in the river based on their importance. Because the number of channels increases when δ decreases, a measure of the importance of a channel is the highest δ value at which that channel still appears in the network. To compute these δ values per channel, we first perform the network computation for a wide range of δ values, say, $\delta_{1}>\delta_{2}>\ldots >\delta_{k}$. This results in k networks, called differential networks, which we then combine into a single composite network (Figure 4). In every network computation, the striation is identical, because the computation of the striation is independent of the value of δ. However, the set of paths selected for inclusion in the network differs. Generally, paths included in the network for $\delta_{i}$ will also be included for $\delta_{i-1}$ and smaller, which leads to significant path overlap when condensing the sets of paths into the composite network. This issue is rectified by a series of postprocessing steps as follows:

1. Channels that are included for multiple δ scales are filtered such that only the largest δ scale at which the channel was detected remains (Figure 4c). Thus, the paths detected by the LowPath algorithm have been converted to network links with starting and ending nodes.
2. In some cases, links at the same scale overlap at certain points in space, which may cause connectivity issues following Step 1. To maintain connectivity, links may be split into smaller sections, and nodes are added at their end points.
3. The channel network is then further segregated into smaller differential networks that detail the nodes and links found at each δ scale (Figures 4d–4g).

After these postprocessing steps, data detailing the coordinates, scales, and the topology of links and nodes are available. An adjacency matrix A is generated for the composite network and the δ differential as a representation of the topology. Geometric information can also be assigned to the links and nodes, such as elevation, channel slope, channel length, or sinuosity.

### 2.4 Data Set

In this paper, we use LowPath and the previously described processing methodology to extract channel network and geometric information from topographic data for both estuaries and braided rivers for analysis and comparison. Both the differential networks and the complete composite network for each data set are analyzed. Four data sets are used: A set of DEMs resulting from the morphodynamic modeling of a braided river (Schuurman et al., 2013), a lidar DEM of the Waimakariri River in New Zealand (Hicks et al., 2007), a set of DEMs from a morphodynamic model of estuary development (Braat et al., 2017), and a DEM of the partially dredged Western Scheldt estuary in the Netherlands (van Dijk et al., 2018, 2019). These data sets were chosen because they span a range of morphological conditions and variability in boundary conditions (i.e., coastal estuaries versus braided rivers). An earlier version of the algorithm was also demonstrated to work for experiments (Kleinhans et al., 2019).

### 2.5 Analysis

A range of statistical metrics have classically been used to describe channel network topology and geometry after the channel network has been extracted. Previous research has largely focused on planform geometries of channels and bars for characterizing the geometry of multithread channels (e.g., and add others Leuven et al., 2016, 2017, 2018; Limaye, 2017). Braiding index or intensity is another commonly utilized metric that quantifies the number of active channels across the width of the channel belt, which we forgo in this paper because it has been addressed and quantified in other studies (e.g., Bertoldi et al., 2009; Braat et al., 2017; Crosato & Mosselman, 2009; Egozi & Ashmore, 2008; Germanoski & Schumm, 1993; Howard et al., 1970; Kleinhans & van den Berg, 2011; Leuven et al., 2016, 2017, 2018; Schuurman et al., 2013). Redolfi et al. (2016) identified the utility of using reach‐scale bed elevation distributions in braided rivers to inform morphological trajectories. However, there lacks information regarding bed elevation distributions within the channel network itself, likely due to limitations in network extraction methodologies. This paper focuses on describing multithread channel networks as a function of channel bed elevation distributions for a range of channel sizes.

For each data set, we analyze the structure of the network at a range of volume parameter values ( δ), measure the distribution of elevations along each link in the network, measure the number of nodes and links at each scale, and calculate the distribution of link lengths for each scale. The elevation distributions are calculated by extracting the elevation in the DEM cell at each coordinate for every link in the network. Cells located at channel confluences and bifurcations are excluded, because these points may bias the results when partitioning the data among the various δ scales. For example, if a small, narrow, and shallow secondary chute channel meets the deep main channel, the elevations at their confluence may significantly skew the elevation distribution of the smaller channel, since the main channel is significantly deeper. Therefore, elevations at these coordinates are excluded when calculating elevation distributions.

Each case study is run at δ scales ranging several orders of magnitude (Table 1). The range of scales is determined by the geometric characteristics of each individual system (e.g., elevation relief, planform extent, and system slope). Since the four case studies chosen range considerably in size, the ranges of δ values are different for each system. However, δ values were selected to ensure that the largest δ scale produced a single main channel, and a simple sensitivity analysis was performed to determine the minimum scale at which this channel is manifested. After the largest δ was determined, δ values were sequentially decreased by 1 order of magnitude until reaching a δ scale that was on the same order as the horizontal grid cell size. Values of δ below this value are physically unrealistic, because channels cannot be detected at finer resolution than one pixel. In Sections 3 and 4, δ is represented qualitatively (from high to low values) rather than quantitatively (actual δ values) for convenience when comparing data sets of significantly different size (see Table 1).

**Table 1:** Summary of the Volume Parameter and Geometric Scales for the Data Set

	δ range	Grid resolution	Average braid belt width
Data set	(m ${}^{3}$)	(m × m)	(m)
Braided River	$3.98\times 10^{2}$ to $3.98\times 10^{9}$	200×80	3,280
Waimakariri	$1.09\times 10^{2}$ to $1.09\times 10^{7}$	8×8	1,050
Estuary Model	$1.20\times 10^{2}$ to $1.20\times 10^{8}$	50×50	2,590 (mouth)–250 (upstream)
Western Scheldt	$1.20\times 10^{2}$ to $1.20\times 10^{9}$	100×100	5,660 (mouth)–2,500 (upstream)

## 3 Channel Network Extraction

**Figure 5:**
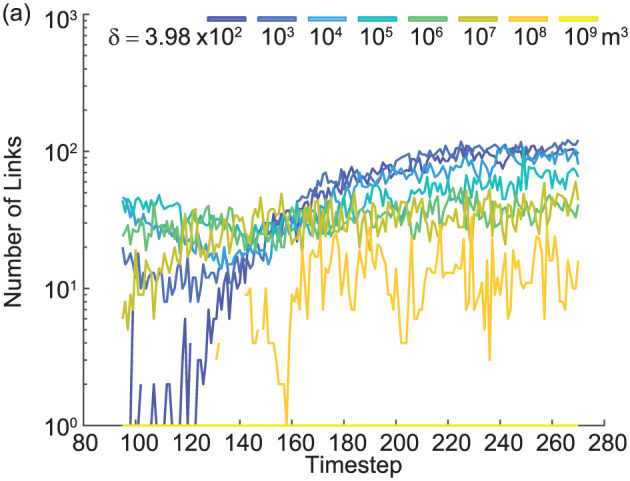
Network change over time for the braided river model of Schuurman et al. (2013). The channel network at time step 180 was chosen for analysis in Figure 6 because it represents the beginning of a relatively steady period of number of nodes and links at each δ scale.

The network structure and geometries are presented for the four data sets discussed in section 2.4. The LowPath algorithm produces channel networks that follow the lowest paths through the topographic surface. Therefore, the extracted network links represent channel thalwegs (the deepest portion of the channel) for the full extent of each channel. For the modeling studies, a representative time step was chosen for analysis based on the changes to the number of nodes and networks likes across δ scales through time (e.g., Figure 5). For example, the braided river model of Schuurman et al. (2013) was determined to be at a dynamic equilibrium state at around model output time step 180 (Figure 5), which marks 12 months of morphological change subject to bankfull flow conditions. The time step was selected because it marked the beginning of a relatively stable period for the number of nodes and links extracted. The same procedure was performed for the estuary model.

**Figure 6:**
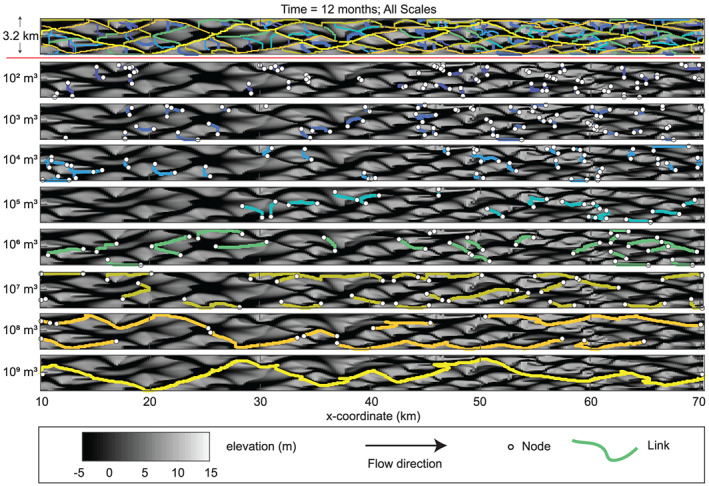
Summary of a multiscale network in the modeled braided river data set. The top panel shows the channel network for a range of scales (network nodes are excluded for visualization clarity). The colors of the channels indicate the volume parameter scale. The following panels show the channel network partitioned by volumetric volume parameter scale. At lower volume parameter scales, channels are relatively short and are often oriented perpendicular to the flow direction. Channels become longer and more parallel to the mean flow direction with increasing scale. The elevation scale is truncated at the lower end for visualization and to match the representation in Schuurman et al. (2013).

Networks are decomposed into differential networks (Figure 6) to isolate the effects of scale on network structure. We use topography from the braided river model of Schuurman et al. (2013) to illustrate these results in Figure 6. At the highest volume parameter scale ( δ), there is one (and only one) lowest path that traverses the landscape from the upstream to downstream boundary (Figure 6). The single link detected at the largest δ scale is representative of the “main” channel of the system. Decreases in δ tend to cause a greater number of channels to be detected, and those channels appear to become shorter in length relative to larger δ scales (Figure 6). In the braided river model (Figure 6), the link detected at the highest δ value (i.e., the main channel) follows an uninterrupted, sinuous path from the inlet to the outlet. The links with the second highest δ value follow a largely similar pattern, but interruptions in the continuity of the links result generally from where these links connect with the highest δ scale link. Discontinuities among the links at a given scale δ are often due to intersections with links at scales greater than the δ of interest.

**Figure 7:**
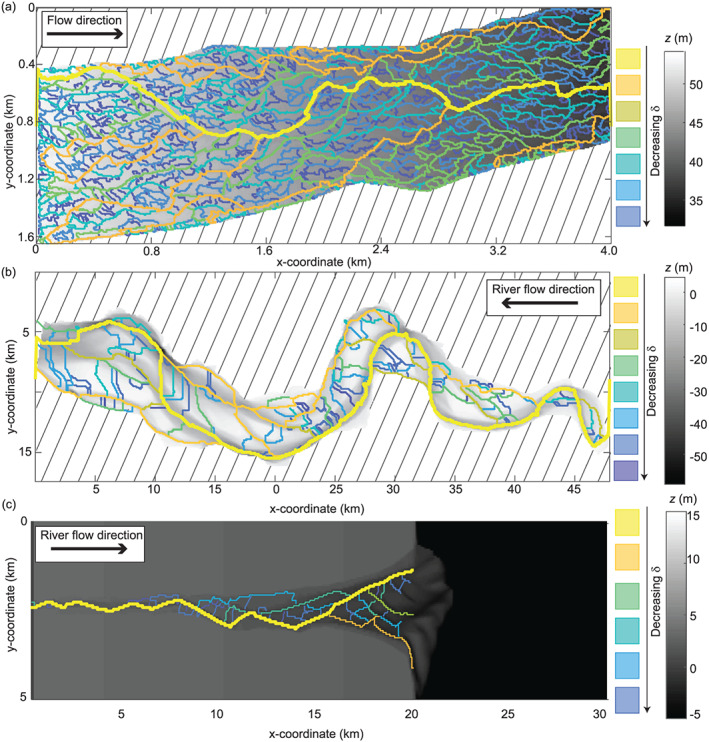
Network extractions for (a) the Waimakariri River (New Zealand), (b) the Western Scheldt estuary (Netherlands), and (c) the results of an estuarine morphodynamics model (Braat et al., 2017). Note the scale exaggeration of the y coordinate of (c) done for visualization purposes. The hashed lines represent areas outside the domain.

Both the channel network of the braided river model (Figure 6) and the channel network of the Waimakariri River (Figure 7a) exhibit a high link density relative to their estuarine counterparts: the Western Scheldt (Figure 7b) and the estuary model (Figure 7c). The estuarine systems tend to have relatively large portions of the channel belt where no links were detected, which is indicative of relatively flat, unchannelized portions of the landscape. These regions vary in size and position within the landscape. By contrast, the links of the braided river systems are uniformly represented throughout the landscape and the unchannelized portions of the landscape have a relatively uniform size and spacing. There does not appear to be a clear spatial clustering associated with the δ value at which channels are detected in the braided river case studies (Figures 6 and 7a), but there appear to be zones of high density of small δ scale channels with bar complexes in the estuarine example of the Western Scheldt (Figure 7b). This behavior is difficult to identify within the estuary model (Figure 7c) because relatively few channels are detected across scales, and the resolution of the numerical model is lower.

Channel bifurcations and confluences are identified during network extractions, and nodes are placed where links bifurcate or join. LowPath maintains the connectivity of these network elements, such that topological information is not lost. The geometric information of bifurcations and confluences is nested within both the elevations at which links and nodes are extracted, but is also manifested in the δ scales of bifurcating or joining links. Notably, most bifurcations involve branches that are identified at different δ values, indicating that the geometry of the two branch channels and the deposited material separating them differ. This indicates that many of the identified bifurcations are not morphologically symmetrical (Figure 7). The tendency of bifurcating channels to be at different δ scales can be seen by decomposing the channel network into separate layers based on δ scale (e.g., Figure 6).

Link length decreases with decreasing δ scale. The relatively deep and wide main channel traverses the extent of the system and is thus significantly longer than those smaller, narrower channels that develop on top of bar surfaces (Figure 6). In between these two extremes, there is a general behavior of increasing link length with increasing δ. This result is expected, since δ is representative of the relative spatial scale of the channel, and larger channels are less likely to be intersected by channels of equal or larger size, and therefore have a tendency to be detected as relatively long and continuous links. This phenomenon holds for all of the cases studied.

## 4 Topology and Geometry

This section presents analyses performed on the extracted networks from section 3 and identifies several topological and geometric characteristics of the studied multichannel systems. The goals of these analyses are to understand how channel network structure varies among different systems and to analyze the extent to which scale influences the internal organization of these channel networks. We present results for the four case studies for which channel networks were extracted with LowPath (Figures 6 and 7).

### 4.1 Number of Links

**Figure 8:**
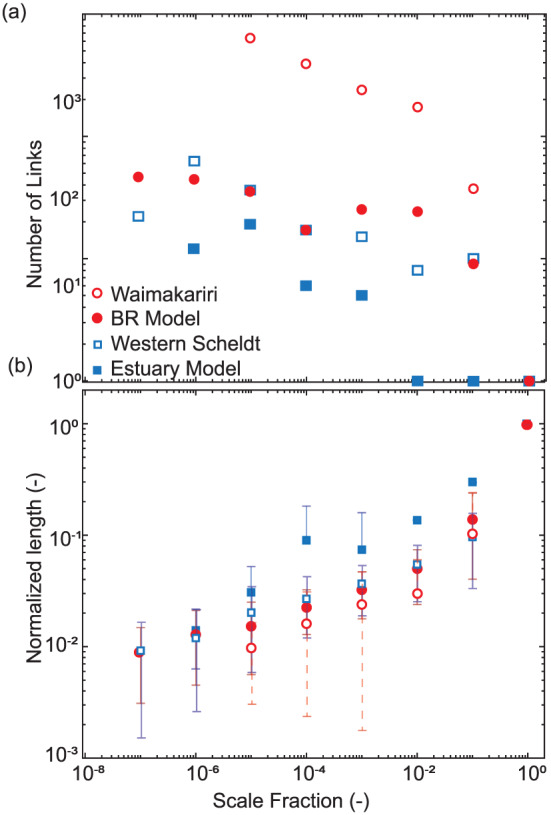
(a) The number of links per scale fraction. (b) The normalized length for links for each data set across the range of delta scales. The sand fraction scales are presented as fractions of the largest scale. The symbols represent the medians of the normalized link length distributions, and the error bars represent the ranges. The mean length of links generally increases with increasing δ. The data appear to follow a power law decay (see text for details).

The number of links in the differential network detected in a given δ scale generally decreases as the scale fraction value increases for each case study (Figure 8a). The Waimakariri has the most links across scales, which is likely due to the relatively high resolution of the topography relative to the width of the braid belt. The estuary model has generally the least number of channel links for a given δ value due to the low number of channels detected. The channel network extracted for the Waimakariri has significantly more links than that of the braided river model (noted as BR model in Figure 8a) and the same is true for the Western Scheldt versus the estuary model (Figure 8a). The difference in number of links at a given δ value between natural and model systems is about 1 order of magnitude.

Within differential networks, the number of nodes detected at a given scale is generally twice the number of links detected at that scale, since a link has a starting and ending node. Multiple links originating from or ending on shared nodes may decrease this total. The inverse relation between node number and δ does exhibit some variability and there are examples where increasing δ values do not cause a decrease in node number. This is likely due to the inherent variability in natural systems and the choice of threshold for δ values. At the upper threshold of δ values there are always two nodes detected for the single “main” channel.

### 4.2 Link Length

The length of each link in the composite network is calculated from the geometric information provided by the topographic surface. For each link i at a given scale δ=j, the normalized length is calculated as

(1) $${\bar{L}}_{\delta =j,i}=\frac{L_{\delta =j,i}}{L_{\text{lowest}}}$$

where L is the length of the link denoted with a subscript i, the subscript j is the delta scale of interest, and $L_{\text{lowest}}$ is the length of the single link extracted at the maximum δ scale (i.e., the lowest path). Likewise, we introduce another normalization to account for the difference in δ thresholds among the case studies. For each case site, the Scale Fraction, is calculated as the scale of interest $\delta_{i}$ divided by the largest sand fraction scale $\delta_{max}$. The values for both Scale Fraction and ${\bar{L}}_{\delta =j,i}$ range between 0 and 1. Performing these normalizations allows for systems of much different spatial scales to be quantitatively compared.

The normalized link length is positively related with scale fraction and appears to follow power law increase behavior (Figure 8b). The exponent on the power relation is 0.23 for the braided river model, 0.27 for the estuary model, 0.24 for the Western Scheldt, and 0.35 for the Waimakariri River. The magnitude of normalized length is mostly similar among the case studies throughout the range of scale fractions considered. However, the estuary model normalized length tends to consistently plot at higher values than those of the other cases, especially at the scale fraction of $10^{-4}$, where the normalized length for the estuary model is nearly an order of magnitude greater than the other three cases.

### 4.3 Elevation Distribution

**Figure 9:**
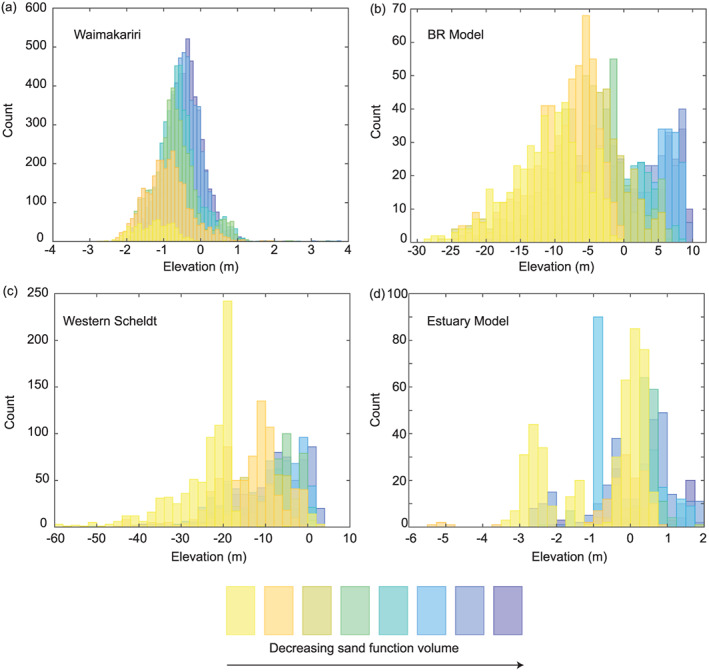
Comparison among the elevation distributions across volume parameter scales for each case study. The elevation values have been corrected for system slope, if necessary. (a) Waimakariri River, (b) braided river (BR) model, (c) Western Scheldt Estuary, and (d) estuary model.

The frequency distributions of slope‐corrected channel bed elevations for the composite network of each case study are displayed in Figure 9. Elevation distributions are constructed by extracting elevation values for each pixel that lies under a link at a given δ scale. The elevation distributions are partitioned into contributions from each δ scale tested to determine how channel bed elevation changes with scale (those classifications are presented qualitatively in Figure 9). In the Waimakariri River channel network, elevations associated with small δ values are generally higher than those associated with larger δ values (Figure 9a). In the Waimakariri River example, this transition from higher to lower elevations as δ increases is fairly gradual which results in a fairly symmetrical, unimodal distribution shape. Additionally, in the Waimakariri River, there are a higher frequency of elevations associated with small δ values. This is due to the large number of channels detected at small δ scales present in the Waimakariri River channel network. Higher δ scales have relatively few and lower elevation values. This pattern of sequentially decreasing elevation with scale is clear for Waimakariri River channel network.

The slope‐corrected elevation frequency distribution of the braided river model channel network exhibits the behavior of decreasing elevations as δ increases (Figure 9b), but the pattern of decreasing frequency in elevation counts from low to high δ values is not present as it is in the Waimakariri River system (Figure 9a). While the overall shape of the elevation distribution appears to be bimodal, the distributions of elevation at each individual δ scale is unimodal. The largest δ scale occupies a large portion of the overall network distribution, which suggests that the main channel is relatively long compared to the cumulative length of channels detected at small scales. However, like the Waimakariri River channel network, the links associated with large δ values are found at lower elevations than those identified at small δ values.

The channel network elevation distributions for the Western Scheldt and the estuary model display different behavior. For the Western Scheldt, the channel network elevation distribution follows a similar pattern of low elevation for high δ values and there is a stark increase in elevation frequency at the largest δ scale around an elevation of z=−20 m (Figure 9c), which is likely due to channel bed maintenance through dredging activities in the estuary. There is also a fairly wide range of elevations at which the largest δ scale link exists. The frequency of elevations is fairly uniform across smaller δ scales in the Western Scheldt. In the estuary model, the elevation distribution for the highest δ scale is bimodal, which is unique among the cases studied (Figure 9d). Additionally, the second highest δ value contains some links, albeit at a very low frequency, with the lowest elevation values around z=−5 m, which again breaks with the general trend observed in the other case studies.

## 5 Discussion

### 5.1 Comparison Among Systems

The novelty of the analyses presented here is the combination of a new network extraction tool for bathymetric data and the comparison between network topology and morphology of fluvial and tidal systems and of field data and numerical modeling. Our results indicate that there are some quantitative similarities between the structure of braided rivers and estuaries for the cases examined in this text.

Visual inspection of our results indicates that the scales of the two channels downstream of a bifurcation are often not the same in the cases studied (see Figures 6 and 7). This result aligns with the evidence that morphodynamically stable bifurcations in most common conditions exhibit asymmetrical partitioning of water and sediment fluxes due to geometric asymmetries between the bifurcate channels (Bolla Pittaluga et al., 2003; Kleinhans et al., 2008, 2013, 2007; Zolezzi et al., 2006). It is reasonable to argue that the geometrical asymmetry associated with the differences in geometry between the bifurcate channels is directly related to the volume of deposited sediment (i.e., channel bar) separating the two channels. Though the discrepancy in scale between bifurcate channels seems to coincide with the literature on bifurcation geometry, the results presented here may be influenced by the calculation of volume within LowPath. In a symmetrical bifurcation, LowPath will still detect assign different δ values to the bifurcate channels. In our analysis, we selected a range of δ values at intervals of 1 order of magnitude to assign scales to channels. This large interval dampens the biasing effects of the LowPath algorithm and increases the likelihood that scale differences are due to geometric discrepancies among channels rather than systematic bias.

Identification of link scale in the form of the volume parameter provides important insight into the stability of bifurcations. However, the stability and functioning of channel junctions in tidal systems are poorly understood, and the network allows testing of theory developed for rivers in tidal systems. Relative channel depths are defining characteristics for river bifurcation stability and discharge asymmetry (Bolla Pittaluga et al., 2015; Edmonds & Slingerland, 2008; Kleinhans et al., 2008, 2013; van Dijk et al., 2014). However, estuaries exhibit mutually evasive ebb‐ and flood‐dominated channels connected at bifurcations, and it is unclear why these asymmetrical bifurcations form with a tidal phase dependence and how this affects propagation of changes through the network (Kleinhans et al., 2015; Leuven et al., 2018; Wang et al., 2002; van Dijk et al., 2019).

The division of channel segments into a range of scales with the physically meaningful unit of sediment volume allows for scaling analysis. Scale invariance and power laws are often used in geomorphology in the search for mechanisms describing system self‐organization and scaling (Dodds & Rothman, 2000; Kleinhans et al., 2005). In network analysis, a scale‐free network is one whose degree (i.e., the number of connections each node has with other nodes) distribution follows a power law distribution with an exponent between −2 and −3 (Albert & Barabási, 2002). There is significant spread in the decay of link count as a function of δ, and the slope of the decay does not follow, in general, a power law decay. Thus, the decrease of link count as δ scale increases (Figure 8a) suggests that the configuration of channel networks in estuaries and braided rivers (i.e., the topology) is not scale independent. This may be expected, since channel networks in nature are chain‐like (Marra et al., 2014), and the connectivity among channels is limited to those in proximity to one another. This causes the network degree distribution to be fairly uniform and cannot follow the power law distribution decay that constitutes a scale‐free network. Conversely, the geometry of the networks suggests some scale‐invariant properties (Figure 8b). The normalized length of channel links increases as a power law with an exponent of around 0.30 for all the cases tested. This suggests that the channel networks multithread channels in both coastal (estuaries) and upland (braided rivers) environments self‐organize in a similar fashion, regardless of size of the system.

**Figure 10:**
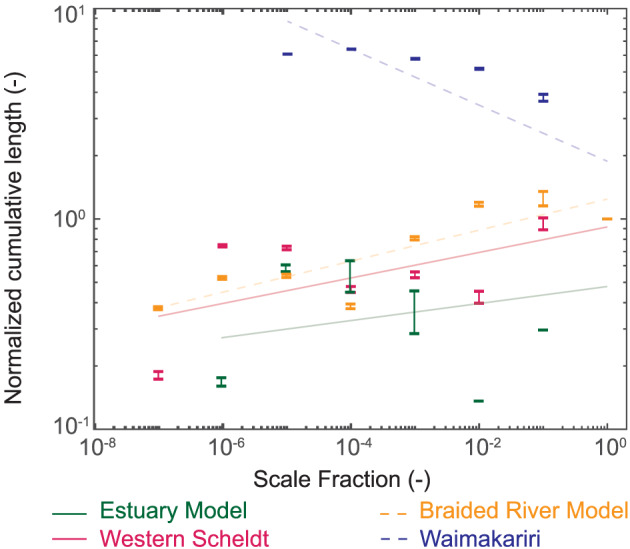
Normalized cumulative length for each tested system with a best fit line included for changes with scale fraction.

The length of channels at various scales obviously depends on the overall length scale of the system in question. In Figure 8b, the length of each network link was normalized by the length of the largest δ scale channel and the normalized length distribution was displayed to compare across systems of different sizes. This metric showed that link length has a rough positive power relation with scale fraction. However, this normalization averages out the effect of the total number of links detected at a given δ scale, which can vary significantly among systems (Figures 6 and 7). To address this, we introduce the normalized cumulative length per δ scale as

(2) $${\hat{L}}_{\delta =j}=\frac{\overset{i=N}{\underset{i=1}{\sum }}L_{\delta =j,i}}{L_{\text{lowest}}}$$

where N is the total number of links at scale δ=j. The normalized cumulative lengths of the braided river model, estuary model, and Western Scheldt systems follow a positive power relation with scale fraction (Figure 10), but has a negative relation for the Waimakariri (Figure 10). The behavior of the normalized cumulative length scale with scale fraction for the Waimakariri is opposite of the trend presented in Figure 8b, while both the normalized cumulative length and the normalized length show similar patterns for the three other systems.

We have two alternative hypotheses for the deviation of the Waimakariri network. First, the much longer collective length of smaller channels than the single main channel may point to an issue of topographic grid resolution. The dependence of extracted channel network features, such as drainage density, on DEM resolution has long been established in catchment hydrology (Ariza‐Villaverde et al., 2015; Garbrech & Mart, 1994; Molnar & Julien, 2000; Sangireddy, Carothers, et al., 2016), and the phenomenon simply depends on the ability of the extraction method to recognize channels; it should recognize smaller channels as grid resolution increases. Many small channels were detected for the Waimakariri system compared to the others (Figure 3), which is likely due to the relatively fine resolution of the Waimakariri lidar used for channel network extraction (Table 1). This results in high cumulative length of channels at small scales relative to the length of the main channel. Thus, for high‐resolution topographies, this result suggests that small scale channels dominate the behavior of the extracted channel network geometry distributions, while systems with lower resolution grids suggest main channel dominance. This may explain the prevalence of bimodality in the elevation distributions (Figures 9b–9d) and lack thereof in the elevation distribution for the Waimakariri (Figure 9a). The second hypothesis is that the larger collective length of smaller channels is a system characteristic. The Waimakariri River is much wider and shallower than the other systems, which leads to a higher braiding index. Regardless of system width, there is only one single main channel with a length of the order of the study reach length, but a higher degree of braiding leads to a higher collective channel length at smaller scales. This hypothesis is supported by the observation that the second largest scale has already a nearly four times larger collective length, and the smallest scales do not become more than a factor 2 higher than that. The second largest scale is not affected by the resolution of the lidar, which argues against the resolution hypothesis.

The elevation distributions (Figure 9) indicated that braided rivers tend to have more overlap among channel elevations across scales (i.e., even large scale channels can be as shallow as small scale ones), but the estuarine systems appeared to have a more bimodel elevation distributions suggesting that a single, main channel tends to develop. Several hypotheses explain these trends. First, this is in qualitative agreement with much higher predicted braiding index in river bar theory than tidal bar theory (Leuven et al., 2016), and also the difference between the modeled and natural braided river is qualitatively expected from their respective channel width‐to‐depth ratios (Kleinhans & van den Berg, 2011). Another possible cause for the deeper estuarine channel is that the natural, midtwentieth century channel depth in the Western Scheldt has been increased by several meters (Verbeek et al., 1998), while the secondary and smaller channel elevations decreased due to dredging for fairway maintenance as demonstrated by modeling compared to controls without dredging (van Dijk et al., 2019). A third hypothesis is that morphological models may have a tendency to erode channels and over‐steepen the bars. However, the estuary model (Braat et al., 2017) was run with a high bed slope effect parameter that prevents such erosion but also subdues bars, changes sediment partitioning at bifurcations and reduces the braiding index (Baar et al., 2019). While this model exhibits bimodality in the elevation distribution, the relatively small number of channels available for extraction at any given time step is likely the source of significant temporal variability in elevation distributions. On the other hand, the braided river model had a much lower bed slope effect and showed runaway erosion of channel beds which caused very deep main channels and relatively steep channel banks, which likely caused the elevations to be unnaturally low at large δ scales. The braided river model also exhibits elevations detected at multiple scales, as in the Waimakariri, because channel bed elevation is not the only factoring determining δ. Bar height and distance between channels also play a role in determining the δ value, so differences in these factors lead to channel elevation being identified at a range of different scales. Finer resolution modeling with between‐channel resolution may be required to adequately compare model results to natural systems. Future work should include topographic resampling to assess the differences/similarities between numerical models and natural systems at equivalent spatial resolutions.

### 5.2 Assessment of LowPath Channel Network extraction

LowPath relies on the geomorphic signatures of the system to identify the channel thalweg in each network link by tracing the lowest elevation paths and is thus insensitive to local bed jumps. The thalweg is an important feature of a channelized system because stream‐wise flow velocities are often highest above the channel thalweg and lateral flow structure is partly dictated by thalweg position and geometry relative to other channel features (Blanckaert, 2011; Konsoer et al., 2016; Valle‐Levinson et al., 2003; Zinger et al., 2013), which drives morphodynamic processes such as point bar deposition, channel bend erosion, chute cutoff (e.g., van Dijk et al., 2012). Clearly, the bed jumps are also important features of channelized systems in relation to the network dynamics.

**Figure 11:**
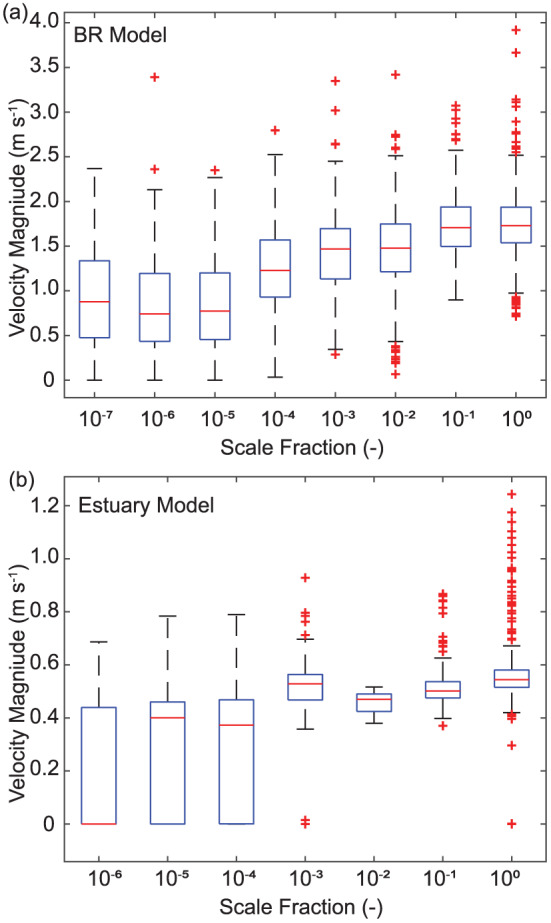
Distribution of velocity magnitude at each model grid cell overlain by a channel link identified by LowPath for the (a) braided river (BR) and (b) estuary models. The data are organized by the δ scale fraction. The central red mark indicates the velocity magnitude median, and the box edges represent the 25th and 75th percentiles, while the whiskers extend ±2.7 standard deviations from the median. Outliers are identified with red crosses.

In numerical modeling efforts, active channels are sometimes identified using a threshold velocity (e.g., Liang et al., 2016) and relative velocity magnitude in space should be a reasonable indicator of channelized flow over a topographic surface. To test the correlation between channel extraction location and the spatial gradients in water velocity, we extracted the velocity magnitude at each pixel that lies under a link at a given δ scale for the same time step as the bathymetery used for channel network extraction for the braided river and estuary model (Figure 11). The estuary model was in ebb flow during the velocity and channel network extraction presented in Figure 11. In general, links identified at small δ values tend to have lower velocity magnitudes than those at larger δ values. Indeed, for both models, the highest median velocity magnitude was identified that the largest δ value, indicating that the link representing the lowest path (i.e., the main channel) had a distribution of relatively high velocities. This result indicates that LowPath identifies channel links that correspond to the primary flow paths in the system. In both of the models tested, the extracted channel network tracks closely with the spatial patterns in velocity magnitude taken at the same time step as the topography used for network extraction, indicating that the extracted links represent primary flow paths over the topo‐bathymetric surface (supporting information Figure S3).

However, in both models, links with relatively small values of δ tend to run perpendicular to the primary longitudinal flow direction (e.g., Figure 6), and those channels tend to correspond to relatively low velocities (Figure 11 and supporting information Figure S3), and sometimes traverse areas with zero velocity. This result is not necessarily unexpected because LowPath identifies the channel network based only on the elevation data and the volume parameter, which allows for the identification of inactive relic channels that are still manifested in the topographic surface, and may also be reactivated. Relatively small channels are also detected by LowPath depending on the selection of the volume parameter and the horizontal and vertical topography data resolution. Channels detected at small δ have limited transport capacity, but may, as part of the braiding dynamics, become cutoffs and large channels. We performed manual extractions of channel networks using the DEMs from the Western Scheldt and Waimakariri River and found that, in general, small scale channels (low δ values near the spatial resolution of the grid) were not identified by user selection but were identified by LowPath (supporting information Figure S4). However, user‐selected channels matched the relatively large scale channels in both cases. LowPath identifies depressions in the topography that are slight and may be challenging to identify with the human eye. Identification of such small scale channels may prove useful for predicting cutoff and braiding dynamics.

The development of methods that track network development through time would allow for advances in model and data analyses. Though LowPath currently extracts channel networks at sequential time steps, each extracted network is independent of the previous time step. This presents a challenge for performing morphological analyses such as tracking the nodal point of a bifurcation through time, assessing avulsions, tracking changes to individual channels, and the classification of active/inactive channels based on morphological development. Further development of channel network extraction methods requires the possibility to define a single multitemporal network structure in both space and time and, for application on discrete data, such rigorous measures for similarity that shifting links and nodes are recognized correctly. In turn, the mathematical rules that correctly identify such shifts require phenomenological models of channel behavior and/or may well capture such natural dynamics.

## 6 Conclusions

This paper presents a method for automatically extracting channel network topology and geometry from multichannel environments using only topography and bathymetry data. The method, called LowPath, relies on extracting the lowest paths traversing a topography across a range of spatial scales, quantified by a new metric for volume‐based channel separation in three‐dimensional environments called the volume parameter. The methodology represents an advancement over steepest‐descent‐based algorithms for detecting channels from topography because those methods cannot handle flow divergences and bed steps, which are ubiquitous in multichannel systems like braided river, deltas, estuaries, and alluvial fans. The new channel extraction method furthers our ability to quantitatively assess channel network structure and geometry in complex environments.

The LowPath method was applied to four case studies: the Western Scheldt estuary, a morphodynamic model of an alluvial estuary, the Waimakariri River, and a morphodynamic model of a braided river. The analyses of the case studies reveal that (1) the number of network links and nodes are inversely related to the volume parameter scale, (2) the relative lengths of links is positively related to the volume parameter scale and this relation follows a positive power law with and exponent of 0.23−0.35, and (3) the elevations of links detected at high volume parameter scales are deeper than those detected at smaller scales. The automatic delineation of detailed channel networks allows fair comparisons between topological and geometrical characteristics of natural systems and those in numerical morphodynamic models. The results suggest that highly braided systems have collectively longer secondary and smaller channel segments than main channel length, as opposed to lower‐braided systems where the main channel has a higher length than the collective smaller channels. Furthermore the results suggest that the tendency to incise channels in the models differs from that in nature for braided rivers and estuaries.

## Supporting information



Supporting Information S1
