# Supplementary material for: Geometry and Topology of Estuary and Braided River Channel Networks Automatically Extracted From Topographic Data
Source: J Geophys Res Earth Surf. 2020 Jan 5;125(1):e2019JF005206. doi: 10.1029/2019JF005206 (PMC7375137; doi:10.1029/2019JF005206)
Supplement: Supplementary file 1 — Supporting Information S1 [file JGRF-125-e2019JF005206-s001.pdf]

Supporting Information for "Geometry and topology of estuary and braided river channel networks automatically extracted from topographic data"

Matthew Hiatt[1,2,3], Willem Sonke[4], Elisabeth A. Addink[1], Wout M. van Dijk[1], Marc van Kreveld[5], Tim Ophelders[6], Kevin Verbeek[4], Joyce Vlaming[1], Bettina Speckmann[4], and Maarten G. Kleinhans[1]

[1]Department of Physical Geography, Faculty of Geosciences, Utrecht University, Utrecht, the Netherlands

[2]Department of Oceanography and Coastal Sciences, College of the Coast and Environment, Louisiana State University, Baton Rouge, USA

[3]Coastal Studies Institute, Louisiana State University, Baton Rouge, USA

[4]Department of Mathematics and Computer Science, TU Eindhoven, Eindhoven, the Netherlands

[5]Department of Information and Computing Science, Utrecht University, Utrecht, the Netherlands

[6]Department of Computational Mathematics, Science and Engineering, Michigan State University, East Lansing, USA

Contents of this file

1. Text S1 to S3
2. Figures S1 to S4

Introduction

The Supplemental Information contains additional information for digital elevation model (DEM) pre-processing before use in LowPath (Text S1), additional explanation of the volume calculation in LowPath (Text S2), and an extended assessment of the validity of the extraction when applied to topography from numerical models and from the field (Text S3). Data files and example scripts necessary to perform the analyses presented in the main and supplemental texts are available in *Hiatt* [2019].

Text S1 - Pre-processing of digital elevation models

In the version of LowPath used for this paper (ver 1.3.6), "source/sink" points are used as the starting and ending points of the path delineation (Fig. S1). All paths identified start and end at a source/sink node. Because of this, DEMs that do not meet the starting and ending nodes obliquely may lead to biased paths due to errors at the boundary. For example, the Western Scheldt digital elevation model (DEM) does not obliquely meet the starting and ending nodes at the links of the domain (Fig. S1). However, on the left side of the DEM, a portion of the channelized network lies near to the starting node. Therefore, paths identified near the links of the DEM may be biased toward channels that are proximal to the starting node, even if there are lower routes that the path "should" take. Thus, the entire set of paths may be affected. Boundary effects can be avoided by padding the DEM with cells of elevation equal to the minimum elevation in the domain (Fig. S1b). This ensures that at the inlet and outlet of the DEM, channel identification is unbiased by the orientation of the DEM.

Text S2 - LowPath volume calculation

LowPath works by identifying the lowest paths in a multi-channel topography and decomposing those paths into a channel network topology. The algorithm identifies the complete set of paths called the striation and calculates the volume separating paths along the isotopy (see main text). Though LowPath considers the total volume separating the channels (throughout the entire DEM), we illustrate the volume separating channels along an idealized cross section (analogous to the isotopy) in Fig. S2. The isotopy considered in LowPath is the one that results in the smallest volume of sediment separating paths. The volume is defined mathematically as the minimum volume above a descending isotopy between the two paths of interest. In other words, the volume separating path p_1 from p_2

is taken as the volume of sediment between p_1 and p_2 that exist at an elevation above the elevation at p_1. This is explained graphically in Fig. S2 where locations A–E represent channels that may be identified by LowPath after thresholding with the volume parameter δ. The volume of sediment separating C and E is V_1, the volume from A to B is V_2, the volume from A to C is $V_2 + V_3$. After the volumes associated with a given path are quantified, the δ scale is used as a threshold for isotopy volumes separating paths. In other words, channels separated by a volume greater than the threshold δ are identified as channels existing at that δ scale. This is repeated for a range of delta values to form the multi-scale channel networks depicted in the main text to ensure that a single δ value is assigned to each link.

Text S3 - Channel extraction assessment

Velocity magnitude can be used to delineate channel networks generated in hydro-morphodynamic models using a threshold velocity to identify active channels [e.g., *Liang et al.*, 2016]. The velocity magnitude should provide an estimate of active channel locations because stream-wise flow velocities will generally be the highest above the thalweg location. We compare the channel network extractions for the braided river and estuary models compared to the model velocity magnitude at the same time step as the bathymetry used for channel network extraction (Fig. S3) and show that there is reasonable agreement between channel link locations and regions of relatively high velocity. The velocity maps and networks presented in Fig. S3 correspond to the velocity magnitude distributions depicted in Fig. 11 in the main text. The estuary channel network was extracted during a ebb tide.

To further examine the capability of LowPath's channel network extraction, we performed channel network extractions by hand using the Western Scheldt and Waimakariri DEMs and compared those channel networks to those identified by LowPath (Fig. S4). The manual extraction was performed by hand using only the topographic information without guidance from the LowPath network. There is good general agreement between LowPath and manual selection for channel detected at relatively large δ, but there is less agreement when δ is small. Generally speaking, channels with small δ values than are oriented perpendicular to the primary flow direction were identified by LowPath but not by manual selection (see x-ccordinates 5 and 25 km in Fig. S4a for examples of this). Accordingly, manual extraction yielded fewer channel links than using LowPath because LowPath is able to identify very slight changes in topography that may not be perceptible to the human eye when inspecting topography.

References

Blanckaert, K. (2011), Hydrodynamic processes in sharp meander bends and their morphological implications, *Journal of Geophysical Research: Earth Surface*, *116*(F1), doi: 10.1029/2010JF001806.

Hiatt, M. (2019), Example input files for LowPath, doi:10.6084/m9.figshare.10324529.v5.

Konsoer, K. M., B. L. Rhoads, J. L. Best, E. J. Langendoen, J. D. Abad, D. R. Parsons, and M. H. Garcia (2016), Three-dimensional flow structure and bed morphology in large elongate meander loops with different outer bank roughness characteristics, *Water Resources Research*, *52*(12), 9621–9641.

Liang, M., C. Van Dyk, and P. Passalacqua (2016), Quantifying the patterns and dynamics of river deltas under conditions of steady forcing and relative sea level rise, *Journal of Geophysical Research: Earth Surface*, *121*(2), 465–496, doi:10.1002/2015JF003653, 2015JF003653.

Valle-Levinson, A., C. Reyes, and R. Sanay (2003), Effects of bathymetry, friction, and rotation on estuaryocean exchange, *Journal of Physical Oceanography*, *33*(11), 2375–2393, doi:10.1175/1520-0485(2003)033¡2375:EOBFAR¿2.0.CO;2.

Zinger, J. A., B. L. Rhoads, J. L. Best, and K. K. Johnson (2013), Flow structure and channel morphodynamics of meander bend chute cutoffs: A case study of the Wabash River, USA, *Journal of Geophysical Research: Earth Surface*, *118*(4), 2468–2487.

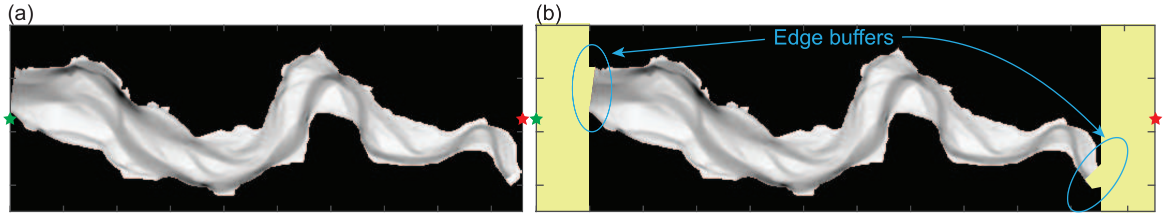

Figure S1. Depiction of edge-buffering to avoid boundary effects in the identification of paths. (a) The example bathymetry of the Western Scheldt (dimensions and scale given in Fig. 7b) and the starting (green star) and ending points (red start) of the lowpath calculation. (b) A cartoon of a "buffered DEM" that pads the beginning and ending regions with the grid cells of the minimum elevation within the DEM (depicted as yellow).

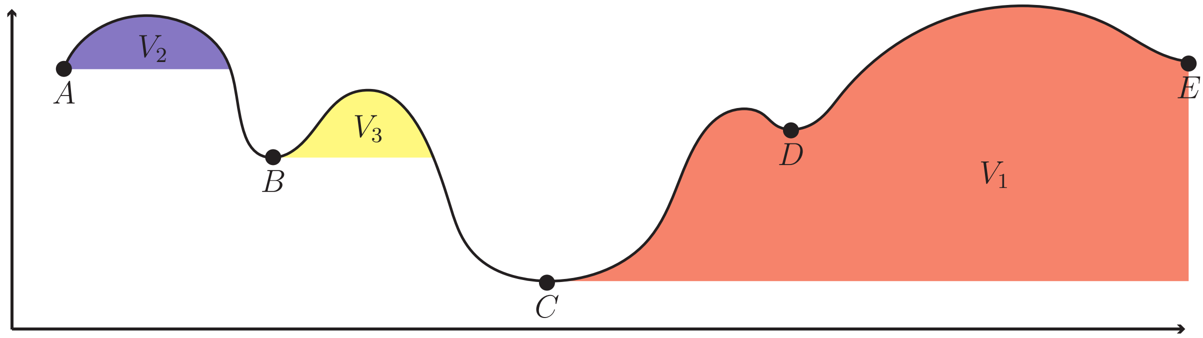

Figure S2. Graphical explanation of the volume separating paths of an idealized topography. The volumes are calculated as the minimum volume of sediment above a descending isotopy that separates the two paths. Locations A–E indicate locations that may be identified as channels and the volumes indicate the volume separating locations A–E. The volume of sediment from C to E is V_1, the volume from A to B is V_2, the volume from A to C is $V_2 + V_3$.

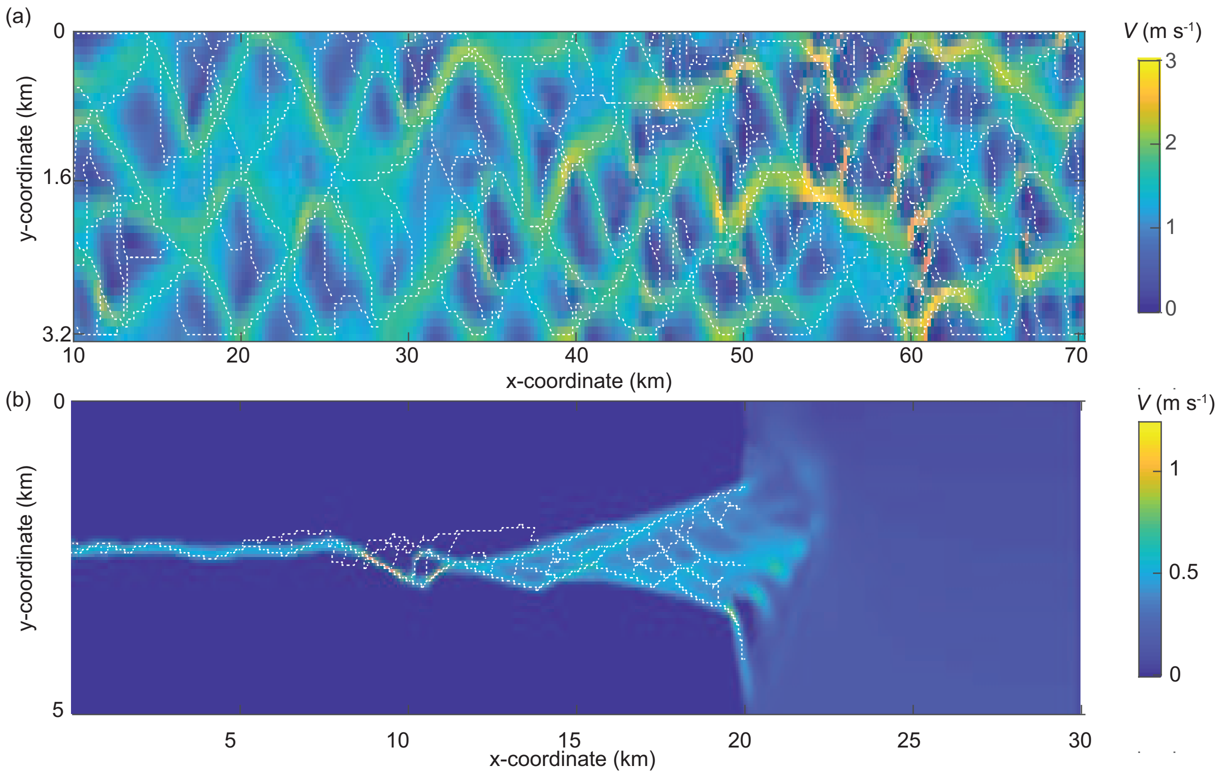

Figure S3. Extracted networks presented as a white dashed line regardless of sediment parameter value overlaying velocity magnitude (V) for the (a) braided river and (b) estuary models. Though the networks were extracted using bathymetric information, the velocity magnitude provides a reference channel network to which the LowPath extraction can be compared. River flow in both models is from left to right. Significant exaggeration in the y-coordinate scale is used for visual clarity that introduces the appearance of discontinuities in the network topology and confluences and bifurcations in the braided river model (a).

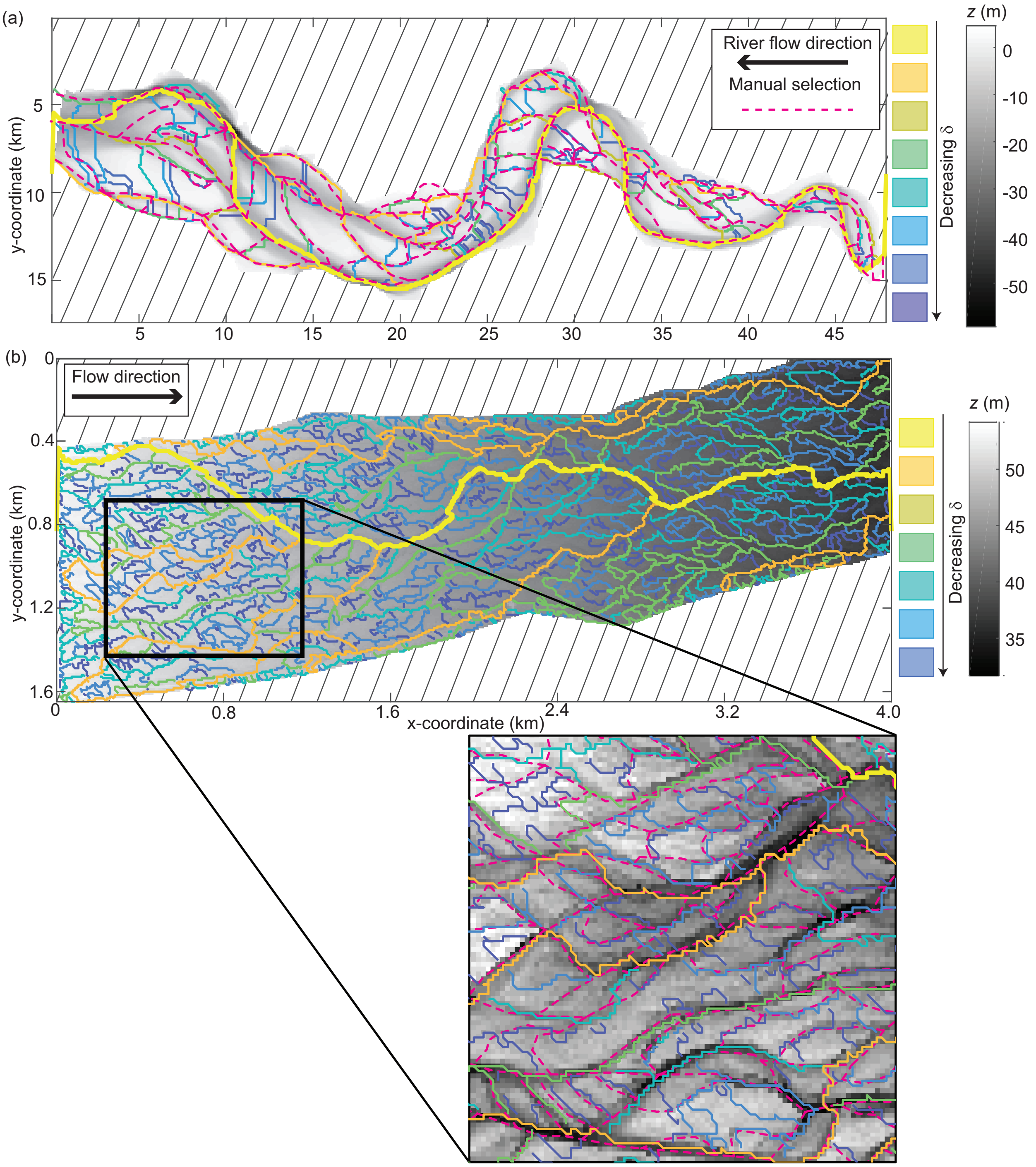

Figure S4. Comparison of the channel networks identified by LowPath and manual selection for (a) the Western Scheldt and (b) a subsection of the Waimakariri River. Only a subsection of the Waimakariri River is shown for visualization, but results were consistent throughout the system.
